# Changes in magnetic resonance imaging disease measures over 3 years in mildly disabled patients with relapsing-remitting multiple sclerosis receiving interferon β-1a in the COGnitive Impairment in MUltiple Sclerosis (COGIMUS) study

**DOI:** 10.1186/1471-2377-11-125

**Published:** 2011-10-14

**Authors:** Stefano Bastianello, Elisabetta Giugni, Maria Pia Amato, Maria-Rosalia Tola, Maria Trojano, Stefano Galletti, Giacomo Luccichenti, Mario Quarantelli, Orietta Picconi, Francesco Patti

**Affiliations:** 1 National Neurological Institute, C Mondino Foundation, IRCCS, Via Ferrata, I-27100, Pavia, Italy; 2 European Biomedical Foundation Onlus, via Nizza 53, 00198, Rome, Italy; 3 Department of Neurology, University of Florence, Viale Morgagni 85, 50134 Florence, Italy; 4 Department of Neuroscience and Rehabilitation, Azienda Università-Ospedale, Corso Giovecca 203, 44100 Ferrara, Italy; 5 Department of Neurological and Psychiatric Sciences, University of Bari, Piazza Giulio Cesare, 70124 Bari, Italy; 6 European Biomedical Foundation, Onlus, Rome, Italy; 7 IRCCS Foundazione Santa Lucia, Via Ardeatina 306, 00179 Rome, Italy; 8 Biostructure and Bioimaging Institute, National Research Council, Via Pansini 5, 80131 Naples, Italy; 9 Opera S.r.l., Via Sampierdarena 33, 16149 Genova, Italy; 10 Department of Neurology, Multiple Sclerosis Centre Sicilia Region, First Neurology Clinic, University Hospital, Via Santa Sofia 78, 95123 Catania, Italy

## Abstract

**Background:**

Conventional magnetic resonance imaging (MRI) has improved the diagnosis and monitoring of multiple sclerosis (MS). In clinical trials, MRI has been found to detect treatment effects with greater sensitivity than clinical measures; however, clinical and MRI outcomes tend to correlate poorly.

**Methods:**

In this observational study, patients (n = 550; 18-50 years; relapsing-remitting MS [Expanded Disability Status Scale score ≤4.0]) receiving interferon (IFN) β-1a therapy (44 or 22 µg subcutaneously [sc] three times weekly [tiw]) underwent standardized MRI, neuropsychological and quality-of-life (QoL) assessments over 3 years. In this *post hoc *analysis, MRI outcomes and correlations between MRI parameters and clinical and functional outcomes were analysed.

**Results:**

MRI data over 3 years were available for 164 patients. T2 lesion and T1 gadolinium-enhancing (Gd+) lesion volumes, but not black hole (BH) volumes, decreased significantly from baseline to Year 3 (*P *< 0.0001). Percentage decreases (baseline to Year 3) were greater with the 44 μg dose than with the 22 μg dose for T2 lesion volume (-10.2% vs -4.5%, *P *= 0.025) and T1 BH volumes (-7.8% vs +10.3%, *P *= 0.002). A decrease in T2 lesion volume over 3 years predicted stable QoL over the same time period. Treatment with IFN β-1a, 44 μg sc tiw, predicted an absence of cognitive impairment at Year 3.

**Conclusion:**

Subcutaneous IFN β-1a significantly decreased MRI measures of disease, with a significant benefit shown for the 44 µg over the 22 µg dose; higher-dose treatment also predicted better cognitive outcomes over 3 years.

## Background

Magnetic resonance imaging (MRI) can provide valuable information on the type, extent and location of pathological lesions in patients with multiple sclerosis (MS). Increasingly, MRI is routinely used in the diagnosis and monitoring of MS, being integral to the McDonald criteria [1]. In addition, MRI measures have been widely used as secondary [2-5] and, more recently, primary [6] outcome measures in large clinical trials of new MS therapies. In the pivotal PRISMS (Prevention of Relapses and disability by Interferon-β-1a Subcutaneously in Multiple Sclerosis) study, subcutaneous (sc) interferon (IFN) β-1a, 44 or 22 µg three times weekly (tiw), significantly reduced relapse-related outcomes in patients with relapsing-remitting MS (RRMS) [7]. Treatment with sc IFN β-1a was also found to significantly reduce MRI measures of disease at 2 years compared with placebo [7]. Significant effects included reductions in T2 burden of disease (BOD; *P *< 0.0001 for both doses vs placebo), the number of T2 active lesions (*P *< 0.0001 for both doses vs placebo), and the number of combined unique active (CUA) lesions (*P *< 0.0001 for both doses vs placebo) [4,7]. Furthermore, a significant benefit of the higher over the lower dose was seen for some measures, including the number of active T2 lesions, as reported for clinical outcomes [7]. Beneficial effects of treatment with sc IFN β-1a on MRI measures of disease were also detected in this patient cohort after 7 to 8 years of follow-up, again with evidence of a dose effect [8].

In addition to long-term monitoring of treatment-related outcomes, MRI, unlike clinical measures, can detect early treatment effects. Recently, rapid benefits of IFN β-1a in RRMS were detected using MRI. Notably, patients receiving active treatment had 69% fewer CUA lesions than those receiving placebo after 16 weeks of treatment (*P *< 0.001) [6]. A *post hoc *analysis revealed a significant effect on the number of CUA lesions as early as week 4 after starting treatment [6]. The effect of IFN β-1a on 'newer' MRI measures including T1 black holes (BH), which indicate areas of axonal loss and permanent tissue damage, is less clear.

Although MRI provides valuable insights into MS, conventional MRI measures have tended to correlate poorly with clinical outcomes [9,10]. This discrepancy is believed to arise for several reasons, such as the presence of clinically silent MRI lesions, functional plasticity, and the failure of neurological measures such as the Expanded Disability Status Scale (EDSS) to capture psychosocial symptoms and cognitive impairment.

Cognitive impairment is experienced by up to 65% of patients with MS [11] and can result in considerable disability, loss of social functioning, and reduced quality of life (QoL) [12,13]. Previous studies in MS have reported associations between the development of brain MRI lesions and cognitive impairment [14,15]. In the prospective, multicentre, observational COGIMUS (COGnitive Impairment in MUltiple Sclerosis) study of mildly disabled patients with RRMS, we reported that T2 hyperintense and T1 hypointense lesion volumes at baseline were significantly higher in patients with cognitive impairment than in those without [16]. Indeed, T2 lesion volume was also found to be a significant predictor of cognitive impairment at baseline [16]. In addition, weak associations were found between cognitive performance test results and specific MRI measures in this population [16].

In COGIMUS, ~20% of patients who received sc IFN β-1a were found to have cognitive impairment at baseline (22 µg sc tiw: 24.2%, 44 µg sc tiw: 18.6% [*P *= 0.145]) despite having only a low level of physical disability (mean [standard deviation; SD] EDSS score was 1.8 [1.0]) [16,17]. Further, 3-year results from COGIMUS suggested that treatment with IFN β-1a, 44 or 22 µg sc tiw, may have a dose-dependent protective effect on cognition in this mildly disabled patient population [17,18]. Here we report the results of a *post hoc *analysis of the longitudinal effects of treatment on MRI measures of disease over 3 years in the COGIMUS study. Associations between MRI measures and cognitive and patient-reported outcomes were assessed to explore whether beneficial treatment effects on MS lesions were related to those on cognition.

## Methods

### Patients and study design

COGIMUS was a prospective, multicentre, observational, 3-year study to assess the effects of IFN-β treatment on cognition in a large cohort of Italian patients with RRMS. The study was performed with respect to the Declaration of Helsinki and according to good clinical practice recommendations (Ethics Committee registration number 78 of the Clinical Experiments Register; 2002, DOI78 Prot. 43 02/08/2002). Full details of the study design have been described elsewhere [16,17]. Briefly, patients aged 18-50 years, with a diagnosis of RRMS (McDonald criteria) [1] and an EDSS score of ≤4.0, and who were naïve to disease-modifying drugs (DMDs), were eligible for inclusion in the study. Patients were assigned to one of three IFN-β treatment regimens: sc IFN β-1a (44 or 22 μg tiw), intramuscular (im) IFN β-1a (30 μg once weekly), or sc IFN β-1b (250 μg every other day); the choice of treatment was made entirely at the discretion of the treating physician following discussion with the individual patient. All patients gave written informed consent. Most patients enrolled into the study (459/550; 83.5%) were assigned sc IFN β-1a treatment. As reported elsewhere [17], 64 patients (11.6%) were assigned im IFN β-1a and 27 patients (4.9%) were assigned sc IFN β-1b, of whom only 13 and 19 patients, respectively, were available for follow-up at 3 years; due to the low patient numbers and high drop-out rates, the current analyses were restricted to the cohort of patients who received sc IFN β-1a. MRI outcomes were compared in patients who received sc IFN β-1a, 44 or 22 µg sc tiw; additional *post hoc *analyses were performed on pooled data from these two patient groups.

### MRI assessments

Standardized MRI scans were performed using a 1.5 Tesla scanner to determine T1 gadolinium-enhancing (Gd+) lesions, and T1 hypointense and T2 hyperintense lesion volumes. Brain MRI scans were performed annually with strict repositioning criteria using standard landmarks. All centres followed a standardized MRI protocol [16]. Briefly, MRI scans were obtained on a high-field magnet. Forty-eight interleaved 3-mm slices were acquired for each sequence, on axial plane; a 250 field of view (FOV) and 256 × 256 matrix were obtained. Specifications included pre-contrast T1: TR 500-650, TE 10-20, one excitation, 6-minute acquisition; post-contrast T1: TR 500-650, TE 10-20, one excitation, 6-minute acquisition; dual-echo sequence: TR 2000-3200, TE 20-50 and TE 80-120, two excitations, turbo factor 4-6, 5-minute acquisition; T2 Fast-FLAIR: TR 6000-9999, TE 150-200, TI 2000-2500, one excitation, turbo factor 11-18, 6-8 minutes acquisition. Scans were analysed centrally by expert observers who were blinded to clinical data.

### Neuropsychological, QoL and psychosocial assessments

Cognitive performance was assessed at baseline and annually up to Year 3. The results of neuropsychological assessments have been reported in detail elsewhere [16]. Briefly, cognitive performance was assessed using Rao's Brief Repeatable Battery and the Stroop Test. Impaired performance was defined as a test result 1 SD below Italian mean normative values [19]; impaired performance on ≥3 tests indicated cognitive impairment [16]. As the number of patients with impaired performance on ≥3 tests and full MRI data was too low for statistical analysis, for the purposes of this *post hoc *study we performed additional analyses to assess the association between outcomes and cognition in which cognitive impairment was defined as impaired performance on ≥2 tests.

Intelligence quotient (IQ) was determined using the Brief Intelligence Test [20]. Fatigue (Fatigue Impact Scale; FIS) [21], depressive symptoms (Hamilton Depression Rating Scale; HDRS [22], social functioning (Environmental Status Scale; ESS) [23] and QoL (Multiple Sclerosis Quality of Life-54 [MSQoL-54] questionnaire) were assessed at baseline and annually thereafter.

### Statistical analyses

Quantitative variables were expressed as means (SD) and medians (Q1, Q3). Discrete and continuous variables were analysed using non-parametric tests: the Mann-Whitney test for between-group comparisons, the Wilcoxon signed rank test for repeated comparisons, and Pearson's chi-square test or Fisher's exact test to compare categorized proportions of patients.

Multivariate logistic regression was used to test associations between MRI variables and clinical and QoL outcomes. Only variables that were significant (*P *≤ 0.05) in univariate models were included in the multivariate model. A clinically relevant change in QoL score was defined as a change of 0.5 - SD or more from baseline to Year 3 (0.5 + SD for the ESS and its subscales) [24]. No adjustments were made for multiple comparisons and there was no imputation of missing data. All statistical analyses were performed using SAS 8.2 and STATA 8.2.

## Results

### Patients and data availability

Baseline MRI data were available for 327/459 (71.2%) patients receiving sc IFN β-1a, for whom MRI data over 3 years of follow-up were available for 164 (35.7%), although all parameters were not available at every time point for all patients. Data were missing for 163 patients either because these patients were lost to follow-up or because the scans were unreadable for technical reasons (e.g. software upgrade, optical disc damage).

Among the 164 patients who had 3-year follow-up MRI data, mean (SD) age was 33 (8) years and 102 (62.2%) were women; mean (SD) disease duration was 5 (5) years and the mean (SD) baseline EDSS score was 2 (0.8). Baseline characteristics were compared in patients with and without MRI follow-up data. No significant differences between these two groups were observed in sex ratio, age, education, QoL, IQ scores (verbal, performance or total IQ) or sc IFN β-1a dose. Significant differences were seen between patients with and without MRI follow-up for: impaired performance on ≥3 tests (12.1% vs 26.5%; *P *= 0.0003); disease duration (mean [SD]: 4.8 [4.9] vs 3.2 [4.1] years; *P *< 0.0001); EDSS score (mean [SD]: 2.0 [0.8] vs 1.7 [1.0]; *P *= 0.0037) and HDRS score (mean [SD]: 8.0 [5.2] vs 6.1 [4.6]; *P *= 0.0002).

### Effect of sc IFN β-1a on MRI outcomes (pooled data)

At Year 3, both T2 lesion and T1 Gd+ lesion volumes were significantly lower than at baseline (Table 1). The mean (SD) absolute decrease in T2 lesion volume was 644.2 (3072.1) mm^3 ^and the mean percentage change was -7.6% (*P *< 0.0001 vs baseline; both measures). T1 Gd+ lesion volume decreased by 48.2 (269.3) mm^3 ^(*P *= 0.0034 vs baseline), and mean percentage change from baseline was -60.5% (*P *< 0.0001). T1 black hole (BH) volume did not decrease significantly from baseline to Year 3.

**Table 1:** Change in MRI parameters from baseline to Year 3 (pooled treatment groups)

	**MRI Parameter**					
	
	**T2 lesions**		**T1 Gd+ lesions**		**T1 black holes**	
	
**Lesion volume, mm^3^**	**Baseline**	**Year 3**	**Baseline**	**Year 3**	**Baseline**	**Year 3**
N	158	158	154	154	156	156
Mean (SD)	5990.1 (7111.6)	5345.9 (6755.3)	73.8 (252.1)	25.6 (125.5)	1263.1 (2430.6)	1185.4 (2520.7)
Median	3378.5	2825.0	0.0	0.0	436.3	341.9
Q1, Q3	1052.9, 7358.5	782.0, 6918.5	0.0, 0.0	0.0, 0.0	76.5, 1189.2	67.9, 1160.0
**Absolute change, mm^3^**						
N	158		154		156	
Mean (SD)	−644.2 (3072.1)		−48.2 (269.3)*		−77.7 (1056.8)	
Median	−135.6		0.0		0.0	
Q1, Q3	−719.8, 86.7		0.0, 0.0		−89.5, 73.4	
**Percentage change^†^**						
N	158		34		132	
Mean (SD)	−7.6 (34.1)^**c**^		−60.5 (149.4)^**‡**^		0.3 (67.8)	
Median	−7.3		−100.0		−4.7	
Q1, Q3	−23.8, 4.0		−100.0, −100.0		−32.1, 19.5	

Gd+, gadolinium-enhancing; IFN, interferon.

*P = 0.0034 vs baseline; Wilcoxon signed rank test.

^†^Percentage change was not calculated for patients with a lesion volume of 0 mm^3 ^at baseline.

^‡^*P *< 0.0001 vs baseline; Wilcoxon signed rank test.

The proportion of patients who were free from new T2 lesions, new T1 Gd+ lesions and new BH at all time points from baseline to Year 3 were: 51.2% (84/164), 83.8% (134/160) and 68.5% (111/162), respectively. When only scans from baseline and Year 3 were considered, 79.9% (131/164) of patients, 93.1% (149/160) and 88.3% (144/163) of patients were free from new T2 lesions, new T1 Gd+ lesions and new BH, respectively.

### Comparison of effects of sc IFN β-1a, 44 and 22 µg, on MRI outcomes

No significant differences between the two dosage groups were seen in T2, T1 Gd+ or BH lesion volumes at any timepoint. The number of new T2, T1 Gd+ and BH lesions did not differ between dosage groups at any time point (Year 1, 2 or 3) nor was there any significant difference in the cumulative number of new lesions over the 3 years of follow-up (data not shown).

**Figure 1:**
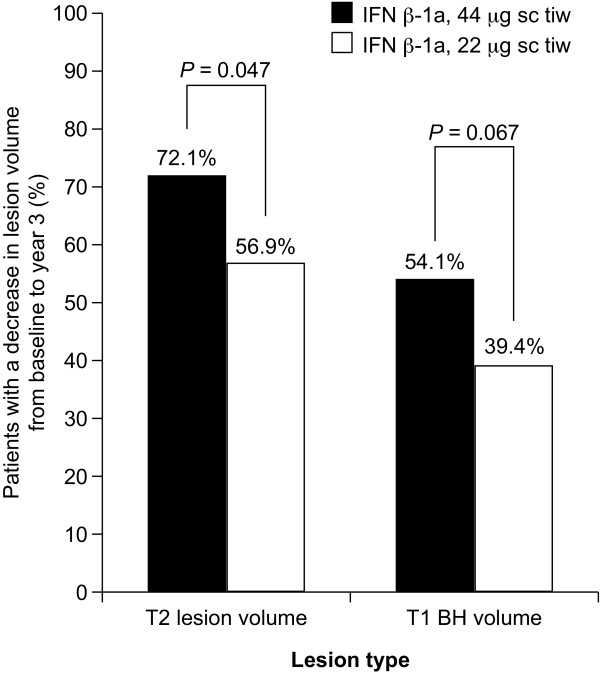
**Proportion of patients with a decrease in T2 lesion volume or T1 black hole (BH) volume from baseline to Year 3, by interferon (IFN) β-1a dose group**. sc, subcutaneously; tiw, three times weekly.

Treatment with IFN β-1a, 44 µg sc tiw, was associated with a significantly greater percentage decrease in T2 lesion volume from baseline to Year 3 than that seen with the 22 µg sc tiw dosage (*P *= 0.025; Table 2). The percentage and absolute change in BH volume from baseline to Year 3 also differed significantly between dosage groups, increasing in the 22 µg group and decreasing in the 44 µg group (percentage change: *P *= 0.002; absolute change: *P *= 0.017 for comparison of doses). There was no significant difference in change in T1 Gd+ lesion volume over time between dosage groups. The proportion of patients who had a decrease in T2 lesion volume between baseline and Year 3 was significantly greater in the 44 µg group (72.1%) than in the 22 µg group (56.9%; *P *= 0.047; Figure 1). A similar trend was seen for the proportion of patients with a decrease in BH volume (44 µg: 54.1%; 22 µg: 39.4%; *P *= 0.067; Figure 1). Multivariate logistic regression showed that treatment with sc IFN β-1a 44 µg predicted a decrease in T2 lesion volume and BH lesion volume compared with the 22 µg dose (Table 3).

**Table 2:** Change in MRI parameters from baseline to Year 3 by IFN β-1a dosage

	MRI Parameter					
	
	T2 lesion volume		T1 Gd+ lesion volume		T1 black hole volume	
	IFN β-1a, 22 μg sc tiw	IFN β-1a, 44 μg sc tiw	IFN β-1a, 22 μg sc tiw	IFN β-1a, 44 μg sc tiw	IFN β-1a, 22 μg sc tiw	IFN β-1a, 44 μg sc tiw
**Absolute change, mm^3^**						
N	72	86	70	84	71	85
Mean (SD)	−174.9 (1609.8)	−1037.2 (3863.3)	−23.5 (198.5)	−68.8 (316.3)	64.5 (608.5)	−196.5 (1311.9)
Median	−81.6	−169.7	0.0	0.0	0.0	−25.3
Q1, Q3	−654.5, 126.8	−1133.3, 13.5	0.0, 0.0	0.0, 0.0	−40.3, 92.5	−209.4, 26.8
P value	0.092		0.472		0.017	
**Percentage change**						
N	72	86	15*	19*	59*	73*
Mean (SD)	−4.5 (27.3)	−10.2 (38.9)	−54.2 (177.5)	−65.4 (127.9)	10.3 (49.3)	−7.8 (79.1)
Median	−3.4	−12.1	−100.0	−100.0	1.5	−16.1
Q1, Q3	−14.5, 4.3	−30.6, 2.8	−100.0, −100.0	−100.0, −100.0	−10.6, 23.2	−50.5, 19.0
P value	0.025		0.495		0.002	

Gd+, gadolinium-enhancing; IFN, interferon; sc, subcutaneously; tiw, three times weekly.

*Percentage change was not calculated for patients with a lesion volume of 0 mm^3 ^at baseline.

**Table 3:** Predictors of cognitive impairment at Year 3, defined as impaired performance on ≥3 cognitive tests (multivariate logistic regression)

	Odds ratio	95% CI
Stable/increased T2 lesion volume over 3 years		
Treatment with sc IFN β-1a 44 µg tiw	0.45	0.23-0.91
Baseline BH lesion volume	1.01	1.00-1.01
Number of new T2 lesions	1.13	1.02-1.26

Stable/increased BH volume over 3 years		
Treatment with sc IFN β-1a 44 µg tiw	0.46	0.23-0.92
ESS score at baseline	0.82	0.69-0.98
Presence of new BH over 3 years	3.21	1.52-6.75

Three or more impaired cognitive tests at Year 3		
Baseline T2 lesion volume	1.01	1.00-1.01
Verbal IQ*	0.92	0.87-0.98
IFN β-1a, 44 μg sc tiw	0.31	0.10-0.98

CI, confidence interval; IFN, interferon; IQ, intelligence quotient; sc, subcutaneously; tiw, three times weekly.

*Higher score denotes superior outcome.

### Cognitive outcomes and MRI measures of disease

Of the patients for whom follow-up data were available at Year 3, 11.8% (18/152) had impaired performance on ≥3 cognitive tests (i.e. were cognitively impaired). The proportion of patients with cognitive impairment was significantly lower in the 44 µg group than in the 22 µg group (6.0% vs 18.8%, *P *= 0.015).

The proportion of patients with a decrease in T2 or BH volume did not differ significantly between those with or without cognitive impairment (*P *= 0.236 for T2 lesion volume; *P *= 0.892 for BH volume). No significant effect of sc IFN β-1a dose was observed.

Patients with ≥2 impaired cognitive tests at baseline were less likely to have decreased T2 lesion volume over the course of the study compared with patients with <2 impaired tests (*P *= 0.034; Table 4). There were no other differences in MRI outcomes over 3 years between patients with <2 or ≥2 impaired tests at baseline.

**Table 4:** Association between changes in T2 lesion volume over 3 years and baseline cognitive impairment

		≥2 impaired cognitive tests at baseline		
			
		No (n = 114)	Yes (n = 44)	*P*-value
Stability or increase of T2 lesion volume, n (%)	Yes	34 (29.8)	21 (47.7)	0.034
	No	80 (70.2)	23 (52.3)	

### Predictors of clinical, MRI and cognitive outcomes at Year 3

Predictors of clinical, MRI and cognitive outcomes at Year 3 were identified using multivariate logistic regression. T1 Gd+ lesion number at baseline was a predictor of being disease-free at Year 3 (odds ratio [OR]: 0.29, 95% confidence interval [CI]: 0.08-0.95). Baseline EDSS score (OR: 0.53, 95% CI: 0.33-0.86), baseline T2 lesion volume (OR: 1.01, 95% CI: 1.00-1.01) and disease-free status over 3 years (OR: 0.31, 95% CI: 0.00-0.84) predicted the development of new BH over 3 years. Baseline T2 lesion volume (OR: 1.01, 95% CI: 1.00-1.01) and new T2 lesions over 3 years (OR: 6.68, 95% CI: 2.09-21.52) predicted the development of new Gd+ lesions. Predictors for the presence or absence of new T2 lesions over 3 years were disease duration (OR: 0.91, 95% CI: 0.84-0.98), and the presence of new Gd+ lesions over 3 years (OR: 4.18, 95% CI: 1.57-11.19).

Treatment with the 44 µg dose of sc IFN β-1a, and higher verbal IQ score at baseline, predicted better cognitive outcomes at Year 3 (Table 3). Higher baseline T2 lesion volume was predictive of cognitive impairment (≥3 impaired tests) at Year 3 (Table 3).

### MRI, QoL and psychosocial outcomes

The proportion of patients with a clinically relevant change in QoL (MSQoL-54 total score only) from baseline to Year 3 was lower in patients who had a decrease in T2 lesion volume over the same interval (12.4%) than in those with stable/increased T2 lesion volume (29.4%; *P *= 0.009). No difference was seen in the proportion of patients with or without a decrease in BH volume. Analyses of fatigue, depressive symptoms and social functioning showed that the proportion of patients with clinically relevant changes in these outcomes did not differ according to whether or not T2 or BH lesion volume decreased over 3 years (data not shown).

Multivariate analyses confirmed that a decrease in T2 lesion volume was a significant predictor of stable MSQoL-54 total score (OR: 0.4, 95% CI: 0.16-1.00). Higher baseline FIS (OR: 1.04, 95% CI: 1.01-1.08) and total MSQoL-54 (OR: 1.08, 95% CI: 1.03-1.12) scores were predictive of clinically relevant changes in MSQoL-54 scores from baseline to Year 3.

## Discussion

The beneficial effects of sc IFN β-1a on MRI measures of disease in patients with RRMS are well recognized. In this observational study, such effects were seen in patients being treated and assessed under normal clinical practice conditions, strengthening our results. Here we demonstrated significant effects of sc IFN β-1a on T2 and T1 Gd+ lesion volumes over 3 years in a large cohort of patients with mild physical disability (mean EDSS score: 2.0). Furthermore, most patients remained free from new T1 Gd+, T2 and BH lesions over the course of the study. A dose-dependent effect was seen in several MRI outcomes, with the 44 µg dose having a more pronounced benefit than the 22 µg dose. Treatment with the 44 µg dose of IFN β-1a also predicted better cognitive outcomes at Year 3. T2 lesion volume was found to be associated with poor cognitive and QoL outcomes.

Over the 3-year study, sc IFN β-1a treatment was associated with a significant reduction in T1 Gd+ and T2 lesion volumes, with a significant benefit of the 44 μg dose demonstrated for T2 lesion volume. These findings are consistent with those of the pivotal PRISMS study, in which sc IFN β-1a reduced T2 BOD and CUA lesion number over 2 years of treatment, with a dose-dependent effect on T2 lesion number and activity [4,7]. Our data confirm that sc IFN β-1a has beneficial effects on MRI measures of disease in this cohort of mildly disabled patients with RRMS. It is interesting that no dose effect was observed for T1 Gd+ lesions. As Gd+ lesions are new lesions, it is possible that the lower 22 μg dose of IFN β-1a is able to inhibit initial inflammatory processes in these patients in the early stages of disease. Indeed, an effect of sc IFN β-1a on active MRI lesions was reported at Month 2 in the PRISMS study, during which patients received lower doses of IFN β-1a during titration up to the 44 µg tiw dose [4]. The dose effect may, however, become evident when assessing longer-term MRI measures such as T2 lesion volume, which capture both old and new lesions. It is possible that newer lesions are more susceptible to the effects of sc IFN β-1a than more established, chronic and inactive lesions.

The presence of BH indicates areas of axonal loss (neurodegeneration). Although we did not find a significant effect on BH lesion volume from baseline to Year 3 in the whole cohort, a significant difference between the two treatment groups was seen regarding the change in BH volume: BH volume increased in the 22 µg group, but decreased in the 44 µg group over the 3-year study. Whether IFN β-1a is neuroprotective is still a subject of some debate; however, our findings may suggest that the 44 µg dose of sc IFN β-1a could have neuroprotective effects. This is a particularly interesting finding as BH were not measured in earlier studies of DMDs, and hence how BH are associated with long-term treatment-related clinical outcomes is not known [8].

At Year 3, only 11.8% of patients had impaired performance on ≥3 cognitive tests. This is slightly lower than the proportion reported in the parent COGIMUS study [17], which may reflect differences between patients who underwent MRI scans and those who did not. Treatment with the 44 µg dose of IFN β-1a was associated with better cognitive outcomes, whereas higher T2 lesion load predicted cognitive decline. An association between T2 lesions and cognitive impairment has been reported previously [16,25-28]. As we saw a clear, dose-related effect of treatment on MRI measures and an association between T2 lesions and cognition, it is interesting to speculate on how the effect on different MRI lesion types may translate into cognitive benefits. Loss of axons (BH) could result in loss of neuronal connectivity, whereas T2 lesions might indicate reduced efficiency of neurotransmission owing to myelin degradation. Mapping the distribution of MS lesions and assessment of those in regions known to govern cognitive processes could provide further insights into how the effects of IFN β-1a treatment on MRI parameters are associated with cognitive benefits. Metabolic studies may also further explain the relation between treatment effects on MRI parameters and cognitive outcomes. In one study using positron emission tomography, the cortical rate of glucose metabolism was shown to be reduced significantly in patients with MS compared with healthy controls, and was inversely correlated with T2 BOD and cognitive performance [29]. White matter lesions could denervate cortical areas and be responsible for the observed reduction in cortical glucose metabolism resulting in clinical symptoms.

Decreased T2 lesion volume also predicted better QoL scores. Associations between MRI parameters (white matter lesion loads and brain atrophy) and QoL outcomes have been described in a cross-sectional analysis of patients with MS [30]. Here, we have assessed longitudinal treatment effects on QoL and MRI disease measures. Together, our and previous observations suggest that treatment-related reductions in MS lesion burden may have a positive effect on patients' daily lives. It is also possible that starting treatment may have positive psychological effects due to the patient's expectation that their treatment will reduce relapses and protect against worsening disability and cognitive impairment that lead to improved QoL. No associations were found between MRI measures and fatigue, depressive symptoms or social functioning in our analysis. However, higher fatigue at baseline did predict worsening QoL. These findings demonstrate the complexity of MS and the importance of measuring both pathological and symptomatic parameters to understand fully the impact of MS on the patient.

The limitations of this analysis should be considered. This was a *post hoc *analysis of data from an observational study, and there was no untreated comparator group; treatment effects should therefore be interpreted with caution. Consequently, differences observed between the lower and higher doses of sc IFN β-1a may have been affected by selection bias, although treatment groups were balanced with regard to main demographic variables. In addition, MRI data at Year 3 were missing for approximately half of the patients with baseline MRI data. Furthermore, among those patients from whom MRI data were collected over 3 years' follow-up, data for some parameters were missing. Despite this, we collected MRI and cognition data for >300 patients at baseline and for >150 patients at Year 3, making this a valuable data set, particularly considering the mild disability and short disease duration in this cohort. Notably, there were some important differences between patients with and without 3-year MRI data, including the proportion who had cognitive impairment, and in EDSS scores. This finding may have influenced the other results reported here and highlights the difficulties that can be encountered with data collection in longitudinal studies. Concerning MRI parameters, we did not assess cortical pathology (inflammatory lesions and atrophy), which has recently been shown to contribute considerably to neuropsychological symptoms in MS [31,32]. As the importance of cortical pathology in MS, particularly with respect to cognitive outcomes, emerged after the start of the COGIMUS study it was not possible to include such assessments in this analysis. In addition, it should be noted that Gd+ lesions can only be detected for a time period of around 1 month at most, so it is possible that some of these lesions may not have been counted due to the scans being performed annually; however, this interval was dictated by routine practice due to the observational nature of the study. Finally, due to low patient numbers, it was necessary to define cognitive impairment as impaired performance on ≥2 cognitive tests to investigate associations between MRI parameters and cognitive status, whereas the definition in the parent COGIMUS study required impairment on ≥3 tests.

## Conclusions

These findings demonstrate that sc IFN β-1a has a beneficial effect on MRI measures of disease in a population of patients with early RRMS and mild physical disability in a normal clinical setting. A dose effect was seen on MRI outcomes, notably on BH, an indicator of neurodegeneration, and was greater with the 44 µg than with the 22 µg dose of sc IFN β-1a. T2 lesion load and treatment with the higher dose of sc IFN β-1a predicted preserved cognitive function over the 3-year study. These results confirm the efficacy of sc IFN β-1a in RRMS and suggest that effects on MRI measures may underlie potential cognitive and QoL benefits of this treatment.

## List of abbreviations

BH: black hole; BOD: burden of disease; CI: confidence interval; CUA: combined unique active; DMD: disease-modifying drug; EDSS: Expanded Disability Status Scale; ESS: Environmental Status Scale; FIS: Fatigue Impact Scale; FOV: field of view; Gd+: gadolinium-enhancing; HDRS: Hamilton Depression Rating Scale; IFN: interferon; im: intramuscular; IQ: intelligence quotient; MRI: magnetic resonance imaging; MS: multiple sclerosis; MSQoL-54: Multiple Sclerosis Quality of Life-54 [questionnaire]; OR: odds ratio; QoL: quality of life; RRMS: relapsing-remitting multiple sclerosis; sc: subcutaneous(ly); SD: standard deviation; tiw: three times weekly

## Competing interests

SB has no competing interests to declare.

EG has no competing interests to declare.

MPA has received personal compensation and research grants from Merck Serono, Bayer Schering, Biogen and sanofi-aventis; and financial support for research activities from Merck Serono, Bayer Schering, Biogen and sanofi-aventis.

MRT has received research funding from sanofi-aventis and compensation for consultancy or speaking from Biogen, sanofi-aventis, Merck Serono and Novartis.

MT has received honoraria for consultancy or speaking from Biogen, sanofi-aventis, Merck Serono and Bayer-Schering; and research grants from Merck Serono and Biogen.

SG has no competing interests to declare.

GL has no competing interests to declare.

MQ has no competing interests to declare.

OP has no competing interests to declare.

FP has received research funding from the University of Catania and Fondazione Italiana Sclerosi Multipla, and personal compensation from Bayer Schering, Biogen-Dompè, Merck Serono and Novartis; and has served on scientific advisory boards for Bayer Schering, Merck Serono, Novartis and Biogen Idec.

## Authors' contributions

SB participated in the design of the study, performed the processing of study data and helped to draft the manuscript. EG performed quantitative analysis of neuroimaging and processing of study data, and helped to draft the manuscript. MPA, MRT, and MT participated in study coordination (patient recruitment) and the collection of clinical data. SG performed quantitative analysis of neuroimaging and helped to draft the manuscript. GL and MQ participated in the design of the study and performed the processing of study data. OP performed the statistical analysis and helped to draft the manuscript. FP participated in the design of the study and study coordination (patient recruitment). All authors read and approved the final manuscript.

## Pre-publication history

The pre-publication history for this paper can be accessed here:

http://www.biomedcentral.com/1471-2377/11/125/prepub
